# Use of dental drill handpiece to remove steel nut causing penile strangulation: a case report and review of the literature

**DOI:** 10.1186/s13256-022-03342-6

**Published:** 2022-04-19

**Authors:** Tuan Thanh Nguyen, Xuan Thai Ngo, Quy Thuan Chau, Khac Chuan Hoang, Le Quy Van Dinh, Hoai Tam Ly, Tien Dat Hoang, Ryan W. Dobbs, Minh Sam Thai

**Affiliations:** 1 Cho Ray Hospital, Ho Chi Minh City, Vietnam; 2 University of Medicine and Pharmacy at Ho Chi Minh City, Ho Chi Minh City, Vietnam; 3 Cook County Health and Hospitals System, Chicago, IL USA

**Keywords:** Penile strangulation, Penile incarceration, Penile entrapment, Dental drill handpiece, Metallic nut, Case report

## Abstract

**Background:**

Penile strangulation is an uncommon urological emergency that requires prompt intervention to avoid potentially serious sequelae including loss of the distal penis secondary to ischemia and subsequent gangrene. We present a case report of a patient who presented to the hospital with penile strangulation injury of 10-hour duration secondary to the presence of a thick hexagonal steel nut. This case is presented in accordance with Consensus Surgical Case Report guidelines.

**Case presentation:**

A 24-year-old Vietnamese man presented to the emergency room with urinary retention and decreased penile sensation following a 10-hour history of penile strangulation due to the presence of a thick hexagonal steel nut that he had placed around the shaft of the penis for the purpose of sexual enhancement during masturbation. The hexagonal nut was tightly entrapping the penile shaft, resulting in edema, congestion, and swelling of the distal 5 cm of the phallus. Given the thickness of the foreign body as well as the degree of penile swelling, we were unable to remove the hexagonal nut using traditional methods of alleviating penile strangulation injuries. Following consultation with a dental colleague, a dental diamond drill handpiece was utilized to cut the foreign body without injury to the underlying penile skin. Subsequent follow-up in clinic demonstrated no significant urinary or sexual sequalae from this episode.

**Conclusion:**

We report a case of penile strangulation requiring novel instrumentation and collaboration for successful treatment.

**Supplementary Information:**

The online version contains supplementary material available at https://doi.org/10.1186/s13256-022-03342-6.

## Introduction

Penile strangulation represents an uncommon urological emergency that was first reported by Gauthier in 1755 [1, 2]. Since then, cases of penile entrapment by a foreign body have been only rarely reported, and only a few case series have been published, with fewer than 100 case reports [1, 3]. Penile rings are utilized by individuals to reduce venous outflow and increase penoscrotal engorgement and may be used by individuals with erectile dysfunction or to enhance sexual gratification. Occasionally, a strangulating object encircling the penis may be associated with patients with an underlying psychosexual disorder [4]. When entrapment occurs, it necessitates urgent intervention since strangulation may cause vascular injury or necrosis, even after removal of the encircling object. Hence, penile strangulation requires prompt intervention to prevent complications [5, 6]. According to the medical literature, management of strangulation penile is also challenging because there is no standard guideline for various conditions, in part due to the heterogeneous nature of such case presentations. Generally, each case is managed individually according to the clinical findings and operative setting [7].

Foreign bodies for penile entrapment comprise many materials, both metallic and nonmetallic. Thin nonmetallic objects are often easy to remove. In contrast, metallic objects are challenging to remove safely. These objects causing penile strangulation in the literature are diverse, including heavy metal rings, hammerheads, metal cones, pipes, plastic bottle necks, sprockets, and plumbing cuffs [8, 9]. Metal objects represent a particularly challenging clinical conundrum as standard surgical equipment in hospital or emergency departments may not be able to cut through these objects.

Furthermore, removing a metallic object is time-consuming, especially thick metallic ones such as hexagonal nuts. Hence, the urologist should be ready and aware of the equipment required for cutting as quickly as possible to manage such medical emergencies. We report herein a case of penile strangulation with a hexagonal steel nut resolved by using an unfamiliar medical tool, viz. a dental drill machine. Our report aims to provide a simple and effective approach to the removal of metallic objects using novel instrumentation to prevent complications such as gangrene and amputation. This case is presented in accordance with Consensus Surgical Case Report (SCARE) guidelines [10].

## Case presentation

**Fig. 1:**
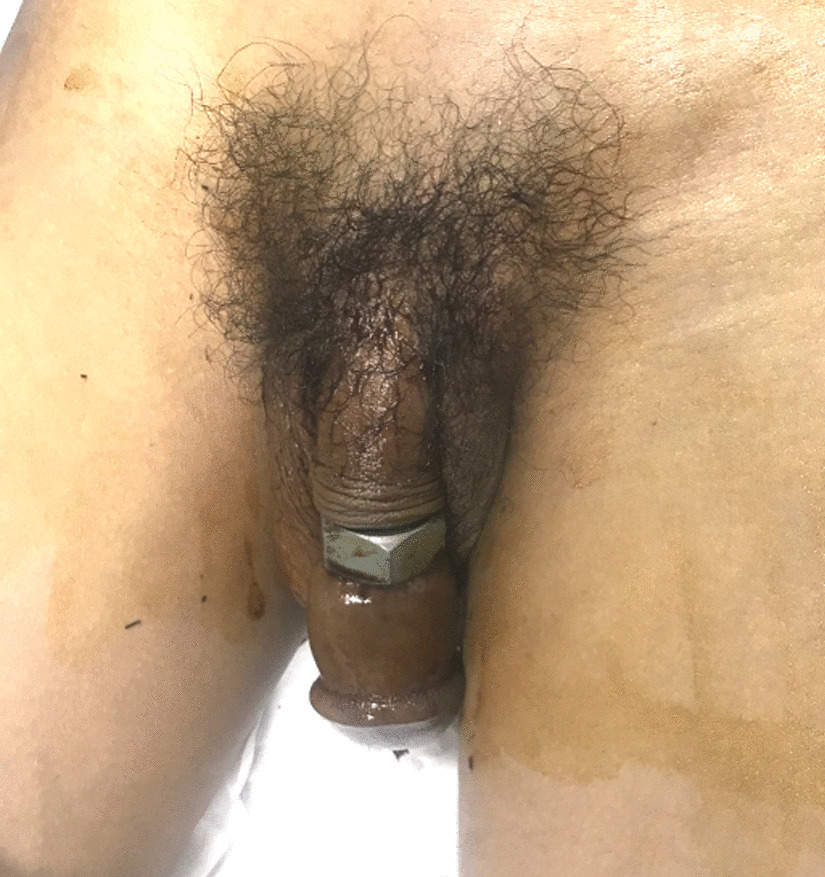
Steel hexagon nut encircling phallus

A 24-year-old Vietnamese man with no significant psychiatric or medical history presented to the Cho Ray Hospital emergency room with penile strangulation of 10-hour duration. Prior to presentation, the patient had placed his penis through a steel hexagon nut for sexual enhancement but was not able to remove the nut after masturbation. On examination, the patient was hemodynamically stable, conscious, and oriented. The patient complained of difficulty with urination and decreased sensation to his genitalia. The patient was uncircumcised, and paraphimosis was present on examination. The metallic nut was located on the penile shaft approximately 5 cm from the distal penis. Physical examination demonstrated that the shaft of the penis, which was distal to the steel nut, was edematous and congested, and the patient reported decreased sensation distally to the entrapping foreign body. There were no signs of necrosis in the glans or distal penile shaft. The initial examination is demonstrated in Fig. 1. This case is typically a grade III penile injury according to the Bhat classification and low-grade injury according to the Silberstein classification (Table 1) [6, 11].

**Table 1:** Summary of grading system for penile incarceration [6, 11]

Grade	Penile injury grading system by Bhat *et al.*	Grading system by Silberstein *et al.*
Grade 1	Edema of distal penis. No evidence of skin ulceration or urethral injury	Low-grade injury
Grade 2	Distal edema, skin, and urethral trauma, corpus spongiosum compression, and decreased penile sensation	
Grade 3	Skin and urethral trauma, no distal sensation	
Grade 4	Separation of corpus spongiosum, urethral fistula, corpus cavarnosum compression, no distal sensation	High-grade injury
Grade 5	Gangrene, necrosis, or complete amputation of distal penis	

**Fig. 2:**
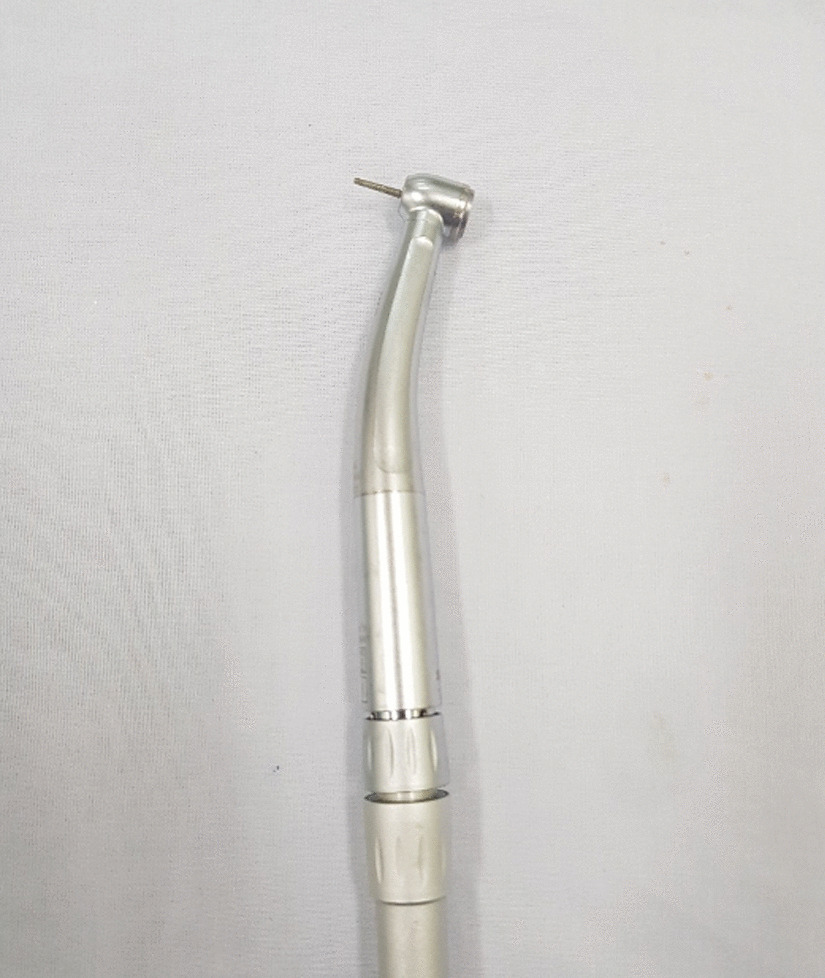
Dental drill handpiece with diamond bur

**Fig. 3:**
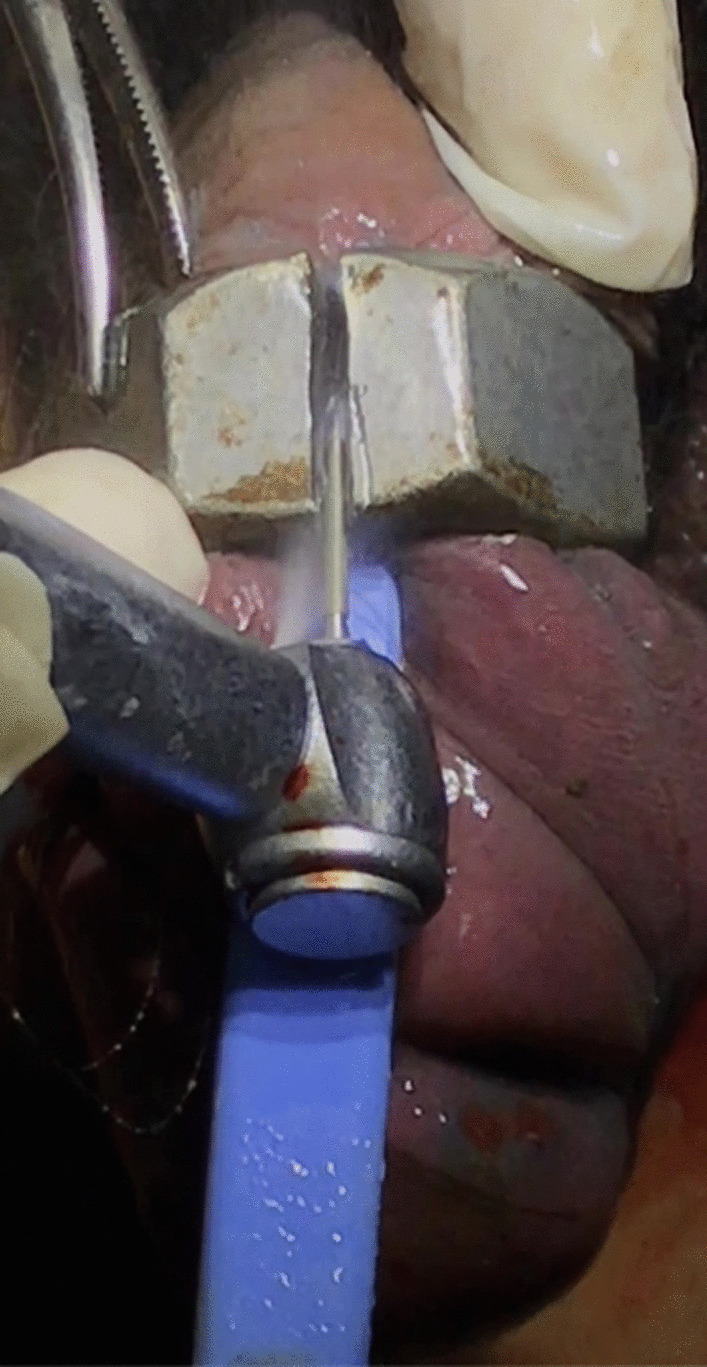
Metal nut cutting with dental drill handpiece

**Fig. 4:**
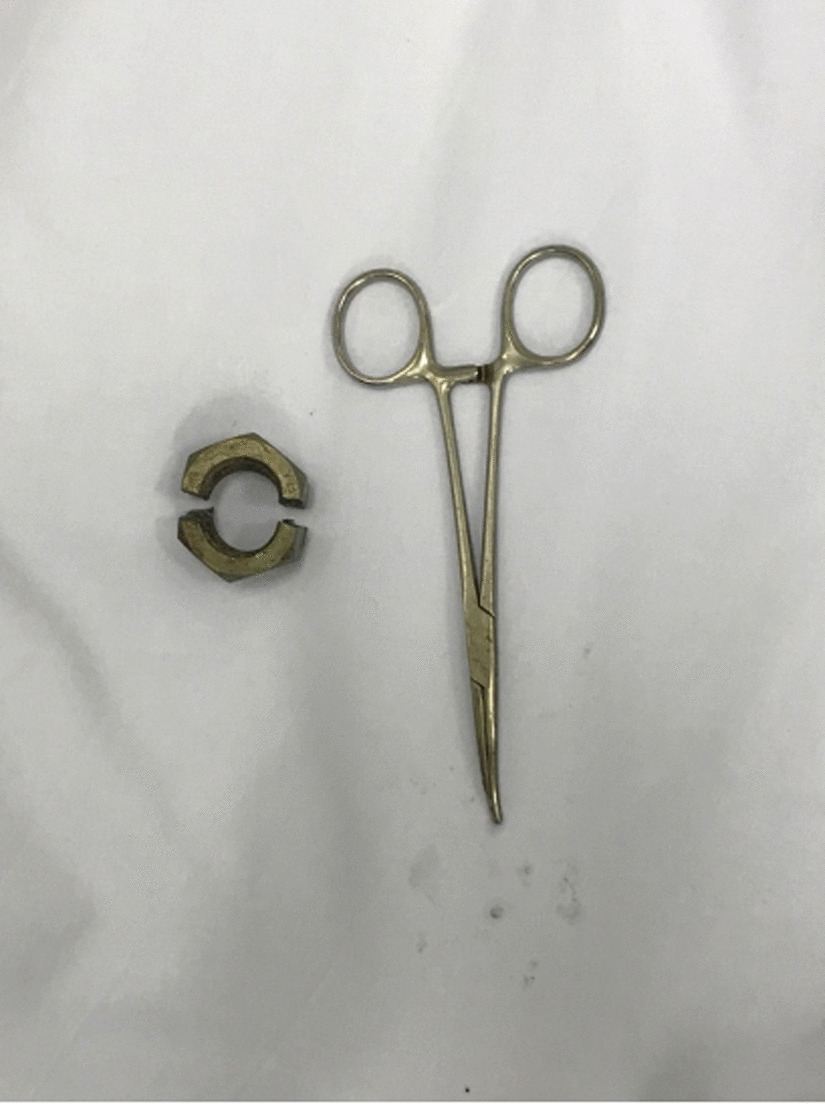
The metal nut is shown following successful removal

**Fig. 5:**
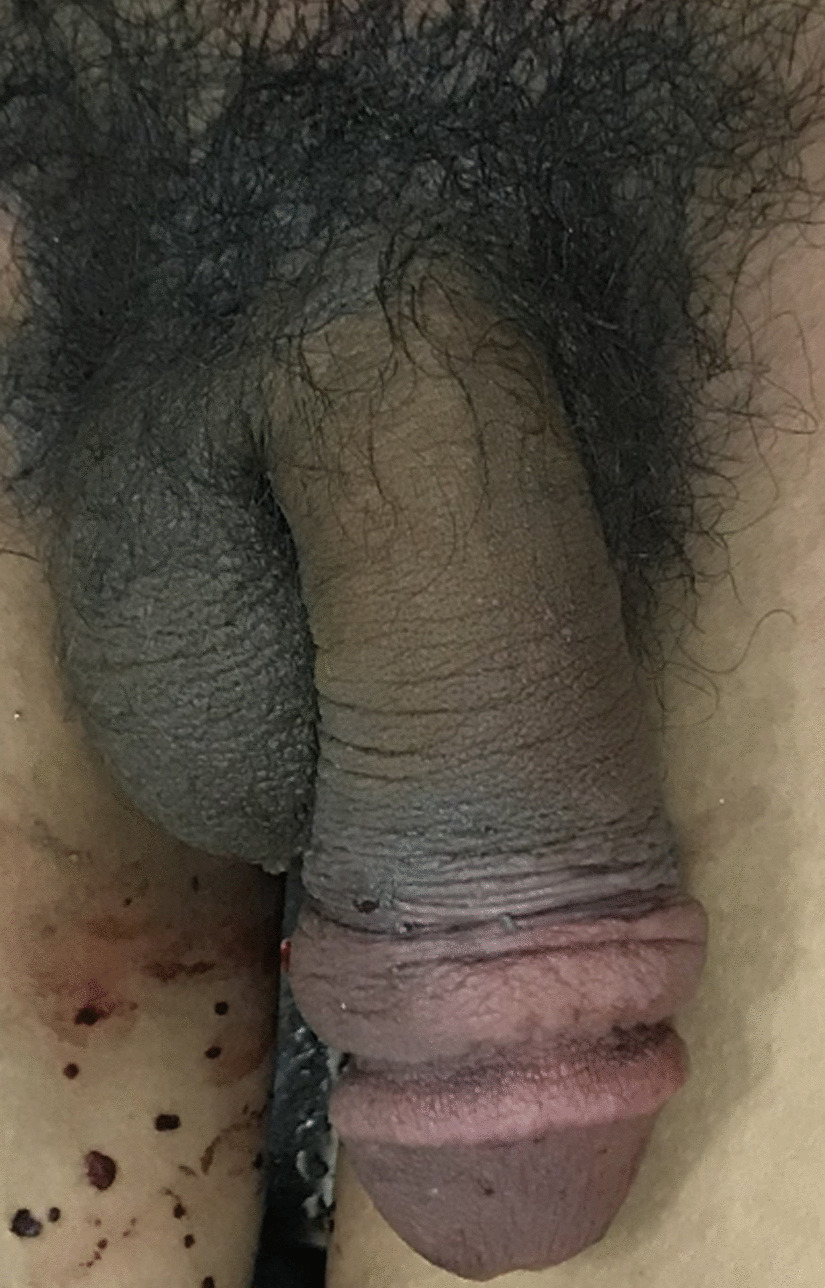
The penis after removal of the metal nut

Following initial evaluation, urgent management placed an intravenous line, and the patient was given analgesics, sedatives, and antibiotics. The patient was not in urinary retention, thus we elected not to attempt to place a urinary catheter. Manual decompression and attempts using lubricant to remove the nut were unsuccessful due to the degree of penile swelling in the distal penis. It was impossible to cut the nut off using a standard bolt cutter as there was no space between the nut and the penile edematous skin. To address this, the use of a dental handpiece was considered, and a dental colleague was consulted by phone. The patient was transferred to the dental clinic in our hospital. The thick metallic nut was removed carefully utilizing a diamond drill in a dental handpiece (Fig. 2). The procedure lasted for approximately 45 minutes with continuous water irrigation to prevent thermal injury to the penis (Additional file 1: Video 1). We used mainly a handheld rotating electric drill to make progress; however, a small plastic blade was also used throughout the procedure to protect the penile skin from the abrasive drill (Fig. 3). After cutting through it at two points, the nut was dislodged from the middle of the penis without damage to the underlying penile skin. The patient was comfortable throughout the procedure. The metal nut measured 2.7 cm in inner diameter, 4.1 cm in outer diameter, and 2.2 cm in thickness; the split nut is shown in Fig. 4 following successful removal. After the nut was removed from the penis, the distal penis was flaccid, the paraphimosis was reduced, and the patient was able to spontaneously void, and the prior distal penile edema and congestion resolved spontaneously (Fig. 5). The patient was placed on antibiotics and analgesics. Psychiatric consultation was obtained to exclude underlying mental conditions or self-injurious behavior. The patient was discharged on day 1 following an uneventful hospitalization. One-month follow-up revealed that the patient had full recovery with normal urinary and erectile function. Erection Hard Score (EHS) obtained at that time was 4/4 [12].

## Discussion

Penile entrapment is a rare urological emergency that can result in significant complications including edema, strangulation, ischemia, gangrene, urethral fistula, and distal penile amputation, particularly when entrapment occurs for longer than 30 minutes [13]. While in our case, removing the nut was done safely in a patient presented after 10 hours of penile strangulation, evidence has shown that the stigma associated with erectile dysfunction and masturbation may contribute to the delayed presentation in most such cases [11]. In adolescents and young men, the most common reason for utilizing these foreign bodies is mainly masturbation and sexual curiosity [14]. On the other hand, middle-aged and older adults use strangulating objects for increasing autoerotic intention and improvement of sexual performance for patients with erectile dysfunction [14, 15].

Strangulation of the penis is always an emergency and may lead to a wide range of vascular and mechanical injuries. Prompt treatment is required, as potential delayed management may lead to complications including vascular obstruction, lymphedema, loss of penile sensation, skin necrosis, urethrocutaneous fistula, urethral injury, gangrene, autoamputation of the penis, and sepsis [16]. Additionally, in such an emergency circumstance, patients are often anxious and fearful given the possibility of significant penile injury. The urologist’s challenge is to relieve the penis of strangulation as quickly as possible to prevent complications. After that, the goals of treatment are decompression and restoration of the penile vascular circulation [14].

In 1991, Bhat *et al.* presented a classification for penile incarceration composed of five grades (Table 1). Subsequently, Silberstein *et al.* simplified the grading system proposed by dividing it into two broad categories [11]. In the Silberstein classification, low-grade injuries correspond to Bhat grade I–III injuries and most of the time require no further intervention after removal of the encircling object. In contrast, high-grade injuries correspond to Bhat grade IV and V injuries and usually require surgical intervention (Table 1) [15]. In 2008, Silberstein *et al.* recognized higher incidence of high-grade injuries in patients presenting after 72 hours (29.1%) in comparison with patients presenting within 72 hours (0%) [11].

The choice of the method for removal of the encircling object depends on its material and size, the incarceration time, the trauma grade, and the equipment available [6, 14]. As the constricting objects involved are variable, physicians must be creative and resourceful because a given technique may be neither applicable nor available in each case. The methods and tools used to successfully remove constricting objects range from aspiration of the corpora cavernosa to the string method, use of saws, orthopedic saws, and industrial pliers [6, 7, 11, 18–21]. Additionally, depending on the entrapment degree and distal edema caused by the encircling penile object, releasing it may be challenging. While the most severe injuries are caused by nonmetallic objects, they can often be easily removed by cutting the constricting object. On the other hand, it may be more challenging to remove metallic objects. A review of the literature to identify different approaches for treatment of penile strangulation caused by metallic objects is reported in Table 2. In our case, we used a dental drill to cut off the metal nut at two sites diametrically opposite to each other for easy removal without iatrogenic injury to the penis. Although dental drills have been used to remove entrapped finger rings, using a dental handpiece as an emergency tool to relieve strangulation of the penis is rare, with only a few documented cases [7, 17].

**Table 2:** Literature review of case reports of penile strangulation caused by metallic objects removed by string technique, nonelectric cutting, and electric cutting devices

Author	Year published	Object	Size	Trauma grade according to Bhat *et al.*	Incarceration time	Treatment method
String technique						
Bucy *et al.* [26]	1968	Ball bearing	2 cm ID 1.5 cm T	2	8 hours	Cord, glans aspiration
Vähäsarja *et al.* [22]	1993	Loop wrench Ball bearing	11 mm ID UKN	UKN 2	5 hours 24 hours	String, glans aspiration String, glans aspiration
Noh *et al.* [21]	2004	Metal bearing Metal bearing	11 mm ID 22 mm OD UKN	UKN UKN	5 hours 8 hours	String, glans aspiration String, glans aspiration
Patel *et al.* [27]	2018	Metal ring (entrapment with both phallus and scrotum) Metal ring	6 cm ID 1 cm T UKN	UKN UKN	24 hours 48 hours	Industrial-grade steel bolt cutters Bolt cutters
Sarkar *et al.* [17]	2019	Metallic plumbing pipe Metal ring Metal ring	4 cm L UKN UKN	2 1 2	6 hours 3 hours 7 hours	Aspiration and string method String method Aspiration and string method
Maregowda *et al.* [28]	2020	Two metal rings	UKN	3	6 hours	String, glans aspiration
Nonelectric cutting devices						
Steiner *et al.* [34]	1978	Metal nut	1 cm W	2	8 days	Hacksaw
Bhat *et al.* [6]	1991	Metal nut Metal nut Metal ring	0.5 cm T 0.5 cm T 0.3 cm T	3 3 2	8 days 5 days 4 days	Hammer and chisel Metal saw Metal saw
Perabo *et al.* [9]	2002	Wedding ring Metal cuff Bull ring	UKN UKN 33 mm W 5 mm T	1 1 1	3 hours Earlier in the day 3 days	Ring cutter Metal saw Bolt cutter
Patel *et al.* [20]	2006	Two metal radiator clamps	UKN	2	6 months	Orthopedic wire cutter
Shukla *et al.* [16]	2014	Metal ring Metal ring	2 cm ID 2.5 cm ID 4 mm T	2 2	14 hours 9 hours	Metal saw Metal saw
Sawant *et al.* [32]	2016	Metal ring	UKN	UKN	4 days	K-wire cutter
Noegroho *et al.* [1]	2021	Metal ring Metal ring Metal ring	UKN UKN UKN	UKN UKN UKN	1 month 18 hours 16 hours	Wire pliers Wire pliers Wire pliers
Electric cutting devices						
Greenspan *et al.* [33]	1982	Steel ring	UKN	2	7 hours	Dremmel moto tool with grinder
Bhat *et al.* [6]	1991	Ball bearing	3 cm T	3	5 days	Heavy drill
Silberstein *et al.* [11]	2008	Metal ring on penis & scrotum	6.5 cm OD 4.5 cm ID	UKN	3 days	Dremmel rotating saw
Etetafia *et al.* [18]	2014	Metal ring	2.2 cm ID	UKN	16 hours.	Dental handpiece
Purnell *et al.* [23]	2016	Two metal cock rings	UKN	UKN	8 hours	Midas Rex Legend pneumatic orthopedic drill
Paonam *et al.* [7]	2017	Metal ring	UKN	3	2 days	Micromotor with wheel shape bur
Low *et al.* [31]	2018	Metal ring	UKN	2	12 hours	GEM ring cutter system with abrasive discs
Ichaoui *et al.* [25]	2018	Metal ring	UKN	UKN	10 days	Angle grinder
Dawood *et al.* [13]	2019	Metal ring	UKN	2	12 hours	Diamond-tipped Midas drill
Agrawal *et al.* [8]	2020	Metal cone ring	0.3 cm T 3 cm W 4.5 cm L	UKN	7 days	Angle grinder
Rahmita *et al.* [19]	2020	Bolt ring	1.5 cm T	3	12 hours	Electric grinder
Kyomukama *et al.* [15]	2021	Metal ring	2.5 cm ID 2 mm T	UKN	72 hours	Angle grinder
Noegroho *et al.* [1]	2021	Metal ring	4 cm W 5 mm T	UKN	1 month	Electric grinder
Present study	2021	Metal Nut	2.5 cm ID 1.2 cm T	3	10 hours	Dental drill with diamond bur

*UKN* unknown, *OD* outer diameter, *ID* inner diameter, *T* thickness, *L* length, *W* wide

Cutting metal produces heat as a byproduct, which may heat adjacent tissues, so care must be taken to cool the metal during this process [7]. The penis must be protected during cutting, which can often be difficult because there is usually little room between the metal and the penis. Likewise, metallic objects must be cut in two spots to avoid damage to the penile skin during removal [23]. In our case, we continuously sprinkled normal cold saline to cool both the metal nut and the penile tissue throughout the drilling procedure. We inserted a plastic tongue-shaped laminar between the strangulating nut and penile skin, which prevented penile skin and tissue injury from the force and heat. The electric dental drill represents an excellent option for removal of obstructing metallic foreign bodies as it cuts very smoothly in a short duration without significant physical exertion. Most importantly for this patient, there are no reported erectile issues after removing the strangulation in short follow-up.

Generally, the management of penile strangulation also depends on the size of the constricting object, incarceration time, injury level, available instruments, and experience of the physicians [6, 14]. If the constricting object is nonmetallic, it can be easily cut off, but thick, hardened-steel or iron nuts are difficult to remove. The lecture review reveals some points for learning:

- Dental or industrial tools can be used to achieve the desired aim of removing metallic objects, especially when there is no space between the nut and the penile edematous skin [11, 17]. In our case, a dental drill was a helpful tool to safely relieve a strangulating penile nut with as little discomfort for the patient as possible.
- More education is necessary to inform users of penile nuts on proper usage and how to prevent strangulation and its complications. After surgical intervention, patients with underlying mental conditions or self-injurious behavior should be referred to a psychiatrist for psychotherapy [4, 5, 24].

## Conclusion

Penile strangulation required emergency management to preserve penile function. A dental drill handpiece may be utilized to successfully remove an encircling metal nut on the strangulated penis of a patient in an emergency.

## Supplementary Information

**Additional file 1.** Video 1: Strangulating penile nut removal.

## Data Availability

The data that support the findings of this study are available from the corresponding author upon reasonable request.
